# Sediment and morphological changes along Yangtze River’s 500 km between Datong and Xuliujing before and after Three Gorges Dam commissioning

**DOI:** 10.1038/s41598-021-93004-2

**Published:** 2021-07-01

**Authors:** Qiancheng Xie, James Yang, T. Staffan Lundström

**Affiliations:** 1 Fluid and Experimental Mechanics, Luleå University of Technology, 97187 Luleå, Sweden; 2 R&D Hydraulic Laboratory, Vattenfall AB, 81426 Älvkarleby, Sweden; 3 Civil and Architectural Engineering, Royal Institute of Technology, 10044 Stockholm, Sweden

**Keywords:** Environmental sciences, Hydrology, Engineering

## Abstract

The impoundment of the Three Gorges Dam on the Yangtze River begins in 2003 and a full pool level is first attained in 2010. This process leads to reciprocal adjustments in flow discharge, sediment transport and morphology downstream of the dam. Based on 26-year recorded hydrologic data 1990–2015 and surveyed bathymetries 1998, 2010 and 2015, this study elucidates, before and after the commissioning of the dam, the alterations along the 500-km reach of the river. Two-dimensional numerical simulations are performed to predict future morphological changes by 2025. The analyses demonstrate that the impoundment modulates the seasonal flow discharges and traps an appreciable amount of sediment, resulting in enhanced erosion potential and coarsening of sediment. On a multi-year basis, the maximum discharge varies by a factor of 1.3 and the corresponding suspended load concentration and transport rate differ by a factor of 3.0 and 3.8, respectively. Combinations of surveyed and simulated bathymetries reveal its morphological responses to the changes. A general pattern of erosion is observed along the reach. In its upper 120 km, the process slows down towards 2025. In the middle 200 km, the erosion shifts, following the gradual impounding, to slight deposition, which then shifts back to erosion around September 2018. In the final 180 km, erosion continues without any sign of de-escalation, which is presumedly ascribed to tidal actions. The reach has not yet achieved a hydro-morphological equilibrium; the riverbed down-cutting is supposed to continue for a while. The combination of the field and numerical investigations provides, with the elapse of time, insight into the morpho-dynamics in the 500 km river reach.

## Introduction

Over the past decades, many rivers have become increasingly fragmented due to the construction of hydropower dams and other wading projects, resulting in major environmental and ecological impacts on the rivers themselves and on the adjacent coastal areas^1–3^. In particular, river damming alters both the flow and the sediment conditions that together modify the conditions in the downstream river course. Consequent to the introduction of the dam in a river, a new long-term equilibrium in the river takes form with time, which incarnates the interplay between the nature and the human beings.

**Figure 1:**
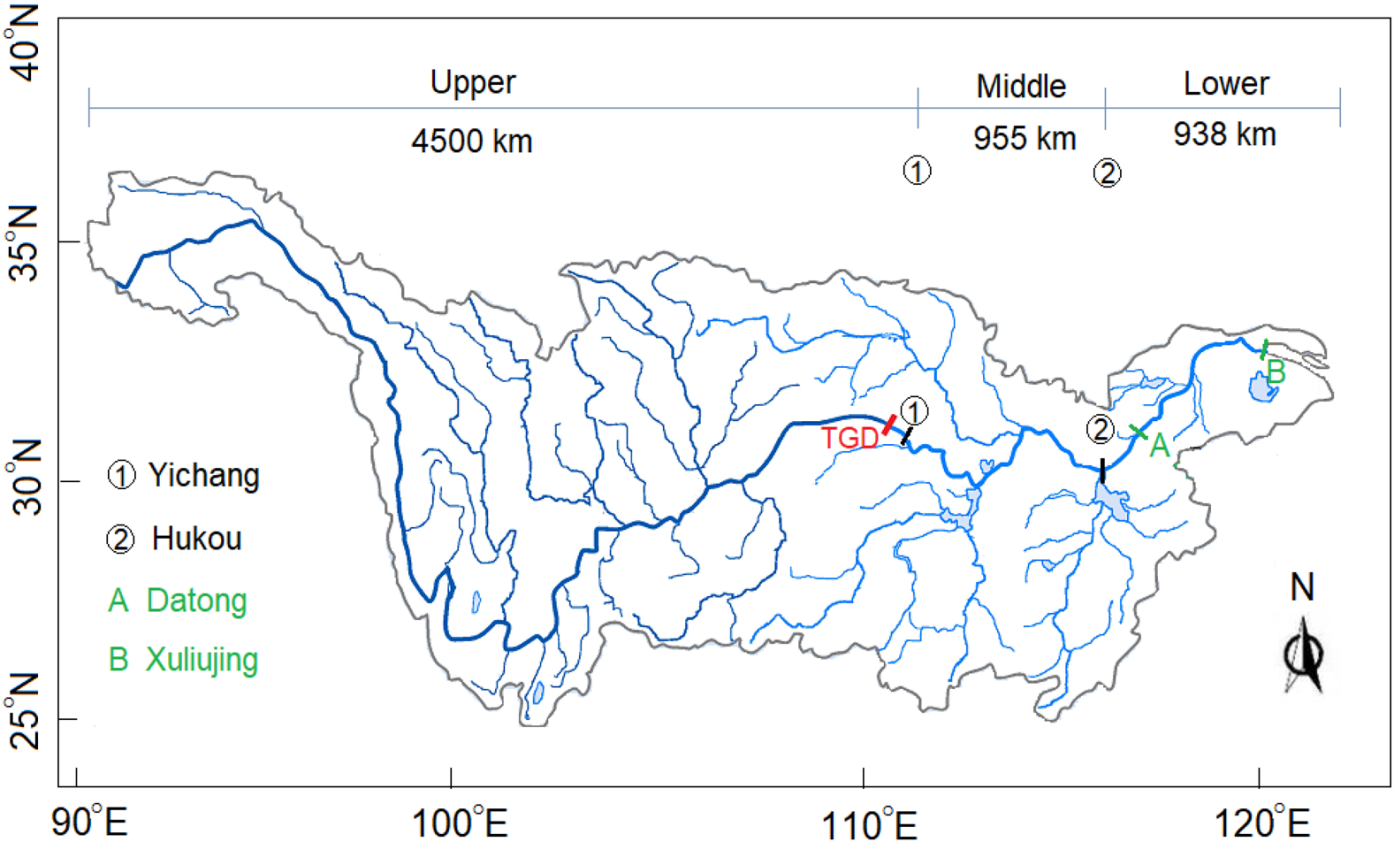
The Yangtze River basin, with indication of the examined reach from the Datong city to the river mouth. Note: the Figure is created using QGIS 3.18 (www.qgis.org) and Adobe Illustrator CC 2018 22.0 (www.adobe.com).

The Yangtze River, with a 1.8 × 10^6^ km^2^ catchment area, originates in the Qinghai-Tibet Plateau (~ 5100 m above the sea level) and flows eastward into the East China Sea (Fig. 1). Globally, it ranks third in length (~ 6300 km), fourth in sediment flux (∼ 470 Mt/year) and fifth in flow discharge (∼ 900 km^3^/year). In the light of the climatical, geological and geomorphological changes, the river is traditionally divided into the upper, middle and lower reaches, the limits of which are at Yichang and Hukou city, respectively (marked by ① and ② in Fig. 1)^4,5^. The upper reach drains the mountainous areas with deep valleys, whereas the middle and lower reaches run through the low-lying plains featuring a wide alluvial water course and many lakes^6^.

The Three Gorges Dam (TGD), located some 45 km upstream of Yichang city, is constructed between 1994 and 2009. Its maximum structural height is 181.00 m; the crest length is 2335 m. The normal reservoir water level is + 175.00 m, at which the water-surface area is 1080 km^2^ and the active storage capacity is 39.3 km^3^. The dam affects the river reach that stretches some 660 km upstream^5^. The total installed turbine capacity amounts to 2240 MW. Table 1 summarizes the characteristic parameters of the dam. The main river closure (the first stage) is accomplished in November 1997. Along with the construction, the reservoir impoundment begins in June 2003 and the first turbine unit starts to generate electricity in July 2003. The water level in the reservoir varies then between + 136.00 and + 143.00 m until October 2006 (initial operation phase). Along with the on-going dam heightening, the water level fluctuates seasonally between + 145.00 and + 156.00 m up to October 2008 (transitional phase). Afterwards, the reservoir level is maintained at above + 173.00 m for approximately 2 years (quasi-normal storage). October 2010 marks the start of the normal operation phase with reservoir levels around + 175.00 m^7^.

**Table 1:** Major dam structural and operation parameters.

Item	Parameter	Value
Dam	Maximal structural height (m)	181.00
	Crest elevation (m a.s.l.)	+ 185.00
	Crest length (m)	2335
Impoundment stages (m)	Initial storage (from June 2003)	+ 135.00
	Transitional storage (from Oct. 2006)	+ 156.00
	Quasi-normal storage (from Oct. 2008)	+ 173.00
	Normal storage (from Oct. 2010)	+ 175.00
Reservoir	*Normal pool*	
	Water-level elevation (m)	+ 175.00
	Active storage capacity (km^3^)	39.3
	Length (km)	663
	Water-surface area (km^2^)	1084
	*Dead storage*	
	Water-level elevation (m)	+ 145.00
	Storage capacity (km^3^)	17.2

Considering the long construction period (∼ 16 years), the reservoir operations have undoubtedly affected the downstream discharges of both water flow and sediment. The downstream impacts have been disclosed in a number of investigations, comprising flood mitigation and control^4,8–11^, subaqueous delta recession^12–14^ and decline of the riverine wetland and lake areas^15,16^. Several of these impacts are caused by the interplay between the water flow and the sedimentation.

As one of the largest dams in the world, the impacts of the TGD on the downstream sedimentation have been an issue of constant concern after its completion^7,10,12,17–20^. Surveys and studies of suspended and bed loads start in 1950 and 1960, respectively. Collected at Yichang hydrological station located some 40 km downstream of the TGD, the field sediment data between 1960 and 1988 show that the sediment yield shifts noticeably from year to year, ranging between 361 and 754 Mt/year. The suspended load averages 523 Mt annually and the bed load only 7 Mt^21^. The dam evens out the inflow, traps most of the bed load and bypasses fine grain sediment inclusive of the suspended load. Yang et al.^7^ analyze the downstream sediment composition, ending up with the same conclusion that the bed load is insignificant in amount. Dai et al.^18,19^ examine the sediment budget to illustrate the sediment dynamics in the lower reaches. They conclude that, shortly after the dam commissioning, both the concentration and flux of the suspended load decline, on a decadal scale, towards a stable level.

As a result of the dam operations and constructions of river regulation works, the lower reaches have experienced continuous channel adjustments^14,22–25^. By a reach-averaged approach, Xia et al.^22^ scrutinize the dynamic adjustments in the bank-full channel geometry of the middle reach after the dam commissioning. Zheng et al.^23,25^ examine the changes in bed elevation and width of the lower reaches, demonstrating channel scouring in most locations. Their studies are based on field bathymetric data from 1998 and 2013, with focus on the impacts of anthropogenic drivers on subaqueous topographical changes. Sediment features and interjacent bathymetry changes are however not unveiled during this relatively long period. Previous studies aiming to predict the downstream channel equilibrium point to the opposite directions. Lai et al.^26^ show, for example, that a new long-term hydro-morphological equilibrium is almost achieved in the lower reaches. In contrast, Yang et al.^7,14^ reveal that, in the coming decades, channel adjustments in form of erosion will continue in response to the sediment reduction, implying that further riverbed erosion is expected.

To shed light upon the sediment and morphological changes incident to the TGD operations, a historical review is first made by a close look at field records during 1990–2015. This includes a pre-construction period, the construction stage with partial commissioning and after the dam completion. The river reach examined covers the last 500 km from Shanghai and up the river. The estuary area is excluded in the study due to its complexity in nature (it is strongly affected by both tides and coastal sediment transport).

For the period of 26 years considered, field flow discharge and sediment data, monthly during 1990–2002 and daily during 2003–2015, are acquired, covering the four project phases. This makes it possible to examine their temporal relationship, so that comparisons are made before and after the impoundment. The river-channel bathymetry is surveyed on three occasions in 1998, 2010 and 2015. With the three sets of bathymetric charts, the riverbed deformation is presented in terms of along- and cross-channel changes. More important to know is the future channel evolution. Two-dimensional numerical simulations of the 500 km reach are therefore also performed to predict the changes in a 10-year perspective. By combining the historical field data and modeling results, the study aims to assess the erosion and deposition patterns along the reach, so that the morphological trend it brings about is well understood. To foresee the near future sedimentation of the large alluvial river has significant implications for the society and the economy.

## Study site

The study site covers the 500 km of the lower reaches of the river, starting at Datong city and terminating at the Xuliujing town (Changshu city), ~ 60 km upstream of the river mouth in Shanghai. Their locations are labelled as A and B in Fig. 1. Datong is situated 1245 km downstream of the dam site. Within the city, the most seaward comprehensive hydrological station of the river is located, denoted as Datong station. This station is the only source of flow discharge and sediment data for the lower river. Hence, almost all published studies dealing with the lower reaches, either local or reach scale, are based on the Datong data. Xuliujing is a gauging station close to the river mouth, which delimits the salty water intrusion into the river. Year 2005 marks the beginning of the automatic tidal current observations.

**Figure 2:**
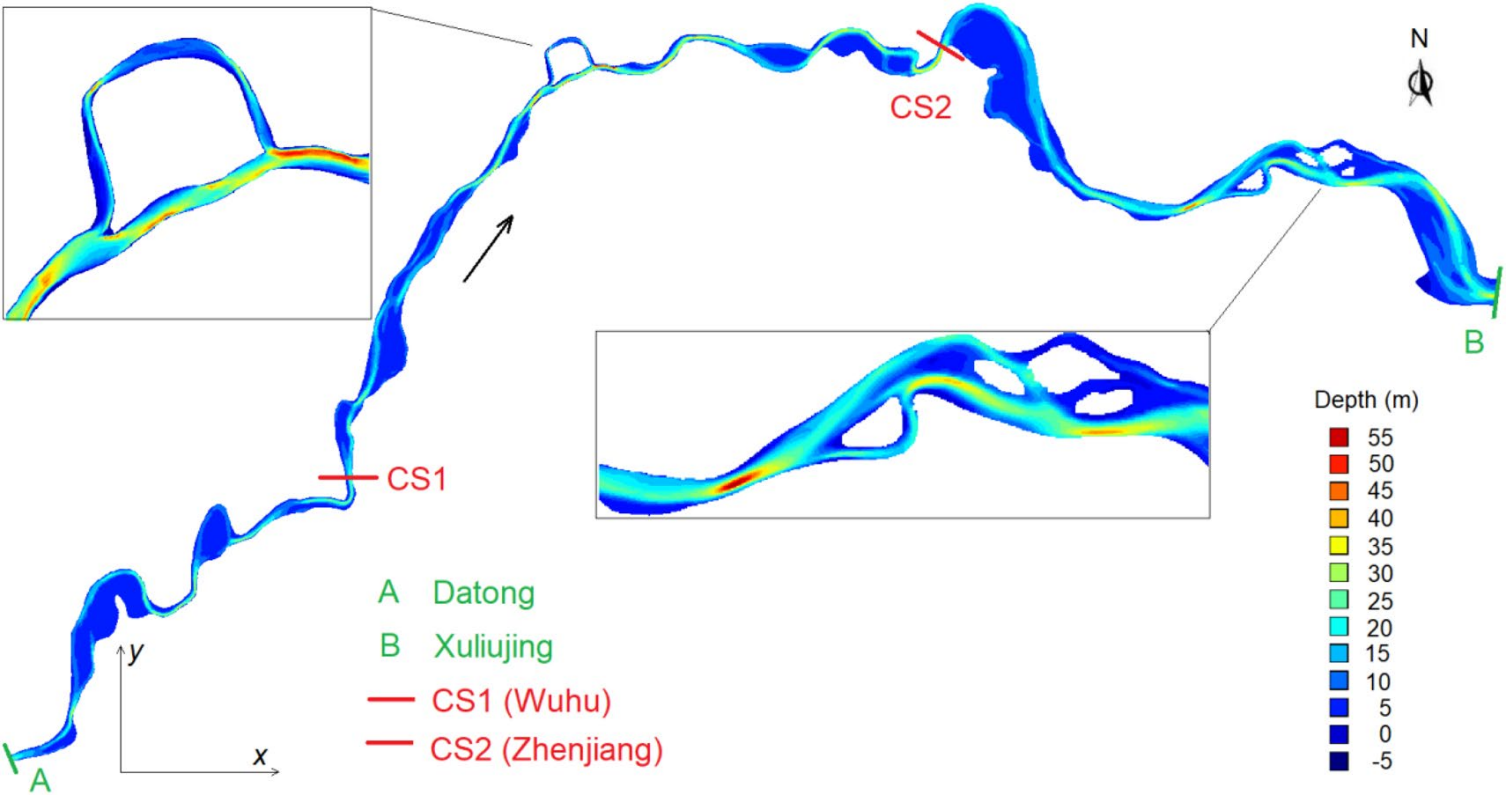
Bathymetry of the examined reach between two hydrological stations (based on field data in 2010).

The reach is characterized by a typical meandering water course with a number of large bends and multi-branches with central bars and islands. Figure 2 shows its bathymetry and also the locations of the two hydrological stations (A and B). It runs through the alluvial plain, with the river bed material composed of medium-fine sand, with a medium size above 0.063 mm^25^. To prevent flooding and bank erosion, levees and various bank revetments are constructed along both sides of the reach. Measured at the normal water level, the average river width and depth are ~ 2400 m and ~ 12 m, respectively^23^. The reach is navigable by ocean-going vessels and it is a major transportation artery, connecting the interior of the land with the coast.

In addition to conveying the flow from its upstream, the river reach also drains ~ 6% of precipitation from the 1.8 × 10^6^ km^2^ river basin area, with an annual mean value of ~ 1200 mm. The region is one of the most densely populated and industrialized areas in China, contributing to ~ 24% of the national GDP.

## Field investigations

### Flow, sediment and bathymetry measurements

At Datong station, the hydrological data of flow discharge and sediment cover the 1990–2015 period and are collected from the Yangtze Water Resources Commission. At Xuliujing station, the water stage and discharge data for the 2005–2015 period are acquired from the Hydrology and Water Resources Survey Bureau of the Yangtze River Estuary. For the study site, i.e. from Datong to Xuliujing, the Yangtze Waterway Bureau maps the river bathymetry on three occasions of concerted operations in 1998, 2010 and 2015, respectively.

For point measurements of flow velocity and suspended load, the procedure is to first position plumb lines along a cross-section (normally 10–30 lines). More lines are placed if the river width is larger. Along each plumb line, measurements of flow velocity and sediment sampling are made at six equidistant positions at *h*_*i*_ = 0, 0.2, 0.4, 0.6, 0.8 and 1.0 of flow depth *H*_0_ (*i* = 1–6) (counted from the water surface). The suspended load is collected with point-integrative water samplers. All the data are recorded at one-hour intervals.

Attached to a customized motorboat with GPS positioning, RDI Workhorse Acoustic Doppler Current Profilers (ADCP) are used to measure flow depth and velocity. At a predetermined transect, the boat goes approximately perpendicular to the direction of the river flow, at a constant cruising speed, ∼ 1.0 m/s. The ADCPs operate in the bottom tracking mode and at the 600/1200-kHz frequency. The four beams are at 20° from each other. The velocity measuring error is ± 5 cm/s; pressure sensors of the YJD-1 type measure water depths, with ± 1 cm accuracy. The relative error of discharge measurements is by estimate below ± 5%. The river bathymetry used in the study is surveyed with HY1600 bathymetric profilers, having a vertical mapping error of ± 1 cm (if *H*_0_ ≤ 20 m) and ± 0.1*H*_0_ cm (if *H*_0_ > 20 m). In addition to cross-sectional flow area and water-level elevation, the measured and derived parameters include flow velocity *V* (m/s), flow discharge *Q* (m^3^/s), suspended load concentration *S* (kg/m^3^), sediment transport rate *Q*_*s*_ (kg/s) and grain-size distribution, all of which are cross-sectionally averaged variables.

To map the flow and sediment in such a large river, challenges do exist, especially during flood seasons, which is ascribed to navigation of the boat to follow the pre-determined transections and maintenance of constant cruising speed. In combination with the river width, high flow velocity and local vortexes are also factors involved. Despite this, measurements are usually repeated 2–4 times to guarantee accuracy. With the elapse of time, the measurement equipment and methods are also upgraded continuously; many manual undertakings are replaced by automatic procedures at the two stations.

### Flow and sediment variations

The TGD modulates both the flow and sediment in the lower reaches, which has a bearing on sedimentation and morphology. Both *S* and *Q*_*s*_ are proxies that are closely associated with the resulting erosion and deposition pattern in the river. Another measure is the grain-size distribution or sediment sorting that can be influenced by the TGD. To understand the morpho-dynamics of the reach, a good knowledge of flow and sediment variations is a prerequisite.

#### Relationship between runoff and sediment flux

**Figure 3:**
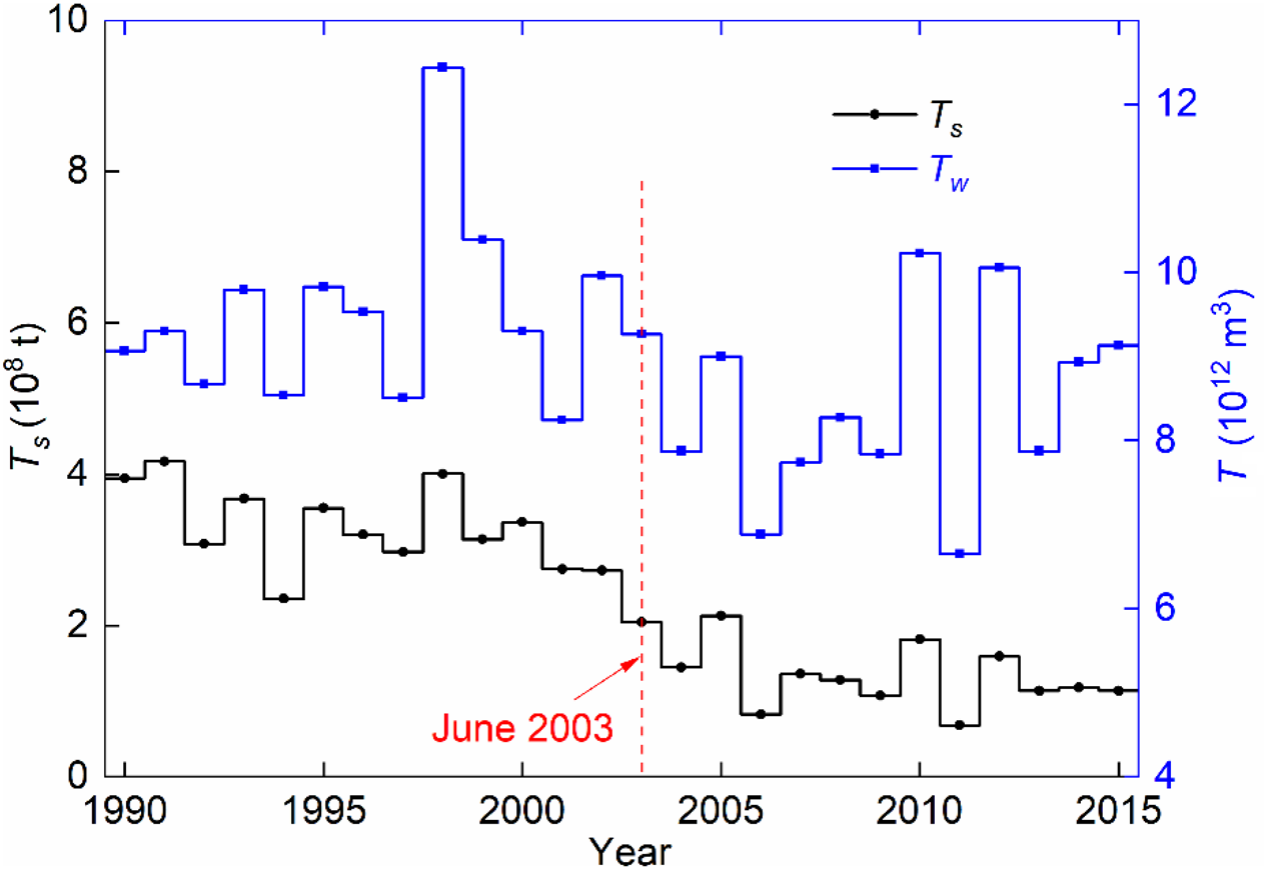
Correlation between annual runoff and annual sediment flux at Datong station during 1990–2015.

Based on the 1990–2015 data series at Datong station, the flow and sediment changes are analyzed. The *Q*, *S* and *Q*_*s*_ data are monthly during 1990–2002 and daily during 2003–2015. As significant amounts of bed load are trapped in the reservoir, relatively clear water is released to the downstream river. On an annual basis, Fig. 3 compares the annual correlation between *T* (m^3^) and *T*_*s*_ (t) before and after the beginning of the reservoir impoundment in June 2003, in which *T* = annual runoff and *T*_*s*_ = annual sediment flux ($\bar{T}$ and $\overline{{T_{s} }}$ refer to their multi-year averages).

The results show that the changes in *T* are not significant except for the wet year 1998 and the dry year 2011. However, a decline is seen after the June 2003 impoundment. For the period 1990–2003, $\bar{T}$ amounts to 9.50 × 10^12^ m^3^; for 2003–2015, it reduces to 8.45 × 10^12^ m^3^, a drop by ∼ 11%. $\overline{{T_{s} }}$ corresponds to 3.30 × 10^8^ and 1.36 × 10^8^ t, respectively. Obviously, the latter is only ∼ 40% of the former. This implies that, following the successive impoundment, *T*_*s*_ exhibits a drastic declining trend.

As the last hydrological station, Xuliujing monitors water stages and other flow parameters, but not the sediment, of the river. Sediment measurements are only carried out in some isolated research projects. Yang et al.^27^ compare, between Datong and Xuliujing, the *S* changes for the 1998–2002 and 2003–2009 periods, which shows good similarity in sediment flux fluctuations with time. In other words, at Xuliujing, its change in sediment budget behaves in a synchronized manner with that at Datong, both demonstrating significant lower levels after June 2003.

Let $\bar{Q}$ (m^3^/s), $\bar{S}$ (kg/m^3^) and $\overline{{Q_{s} }}$ (kg/s) denote multi-year averaged values of *Q* (m^3^/s), *S* (kg/m^3^) and *Q*_*s*_ (kg/s). To look into the monthly variations in flow and sediment, Table 2 presents the averaged results of the same month during the whole examined period (1990–2015). During the years, $\bar{Q}$ and $\bar{S}$ amount to 28,331 m^3^/s and 0.274 kg/m^3^, respectively; $\overline{{Q_{s} }}$ is equal to 9669 kg/s. Obviously, the monthly distribution of each variable is uneven over the period. To exemplify, the fraction flow rate from June to September is ∼ 51% and merely ∼ 18% from December to March. The corresponding value of sediment flux is ∼ 71% and ∼ 5.6%, respectively. Also, note that $\overline{{Q_{s} }}$ has its maximum in July with 28,495 kg/s and its minimum in January with only 1097 kg/s, i.e. the ratio of maximum to minimum is > 26.

**Table 2:** Multi-year averages of flow and sediment of the same month during 1990–2015 at Datong station.

Month	$\bar{Q}$(m^3^/s)	$\bar{S}$(kg/m^3^)	$\overline{{Q_{s} }}$(kg/s)
Jan	13,060	0.084	1097
Feb	13,781	0.081	1116
March	18,790	0.129	2424
Apr	24,245	0.156	3782
May	31,940	0.208	6644
June	40,731	0.318	12,952
July	50,975	0.559	28,495
Aug	44,217	0.523	23,125
Sept	38,156	0.488	18,620
Oct	28,281	0.407	11,510
Nov	20,635	0.216	4457
Dec	15,164	0.119	1805
Average	28,331	0.274	9669

To further categorize the temporal variations, the 1990–2015 period is broken up into four phases:

phase I: January 1990–December 1994, pre-construction period;

phase II: January 1995–May 2003, construction stage and before reservoir impoundment;

phase III: June 2003–September 2010, start of progressive impoundment and continued construction to August 2009;

phase IV: October 2010–December 2015, full pool operation. The reservoir attains, for the first time, its full pool level in October 2010.

**Figure 4:**
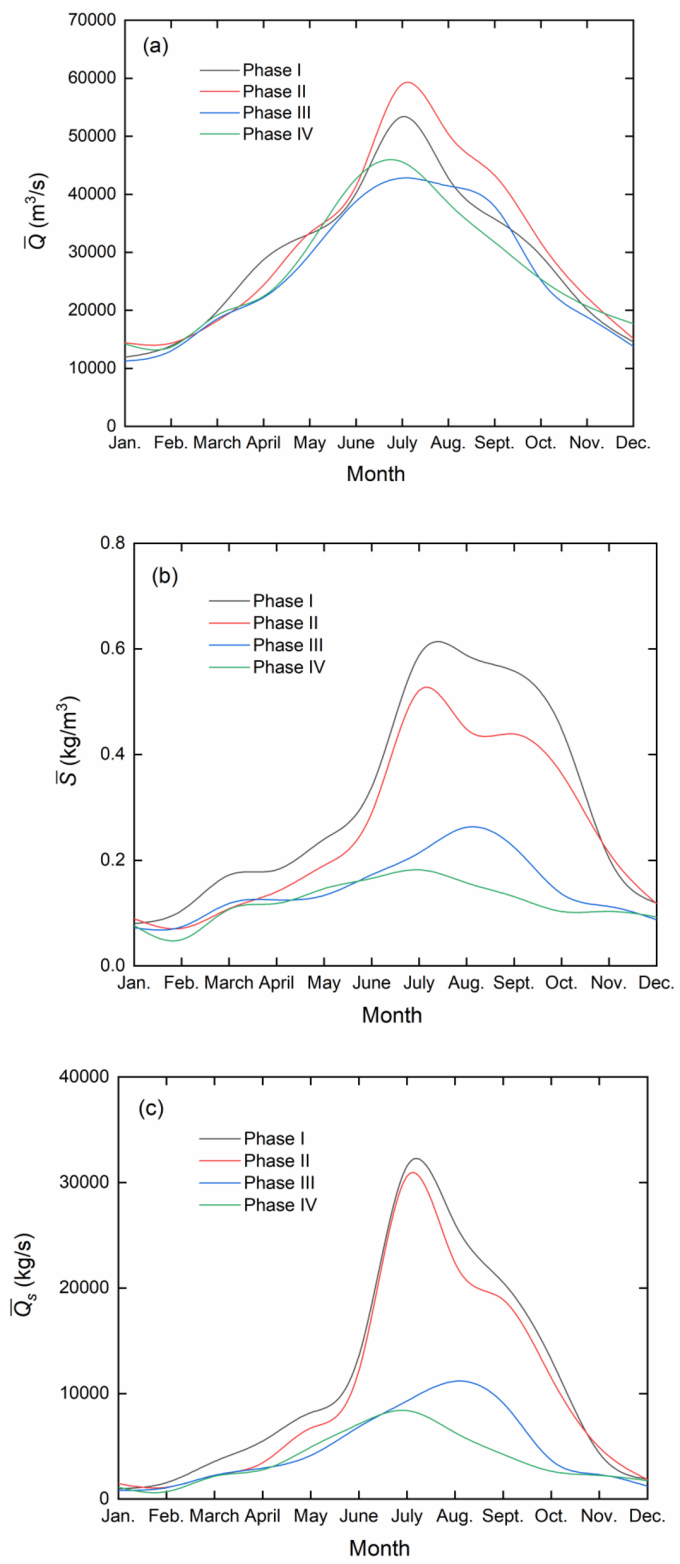
Monthly variations in $\bar{Q}$, $\bar{S}$ and $\overline{{Q_{s} }}$ for each of the four phases (1990–2015) at Datong station. (**a**) ${\text{~}}\bar{Q}$; (**b**) $\bar{S}$ and (**c**) ${\text{~}}\overline{{Q_{s} }}$.

Figure 4 presents, for each of the four phases, the monthly variations in $\bar{Q}$, $\bar{S}$ and $\overline{{Q_{s} }}$. Apparently for phases I and II, each parameter has an uneven monthly distribution and its peak occurs mostly during the July and August months, corresponding usually to the flood season of a year. $\overline{{Q_{s} }}$ follows, by and large, the change in $\bar{Q}$ and is slightly modified by $\bar{S}$. The reservoir impoundment (phases III and IV) damps the flood along the lower reaches and leads to a lower $\bar{Q}$, especially after the summer flood season. The peaks of $\bar{S}$ and $\overline{{Q_{s} }}$ also drop dramatically and become smeared out. In phase III, following the $\bar{Q}$ variations, $\bar{S}$ and $\overline{{Q_{s} }}$ have their peaks during August and September. Xu et al.^28^ analyze, during 2003‒2006, the monthly variations in $\overline{{Q_{s} }}$, demonstrating that most of the sediment is trapped primarily during the high flood months. The timing of the $\overline{{Q_{s} }}$ peak shifts from July to August or September. In phase IV of the normal operation, the changes in $\bar{Q}$, $\bar{S}$ and $\overline{{Q_{s} }}$ are similar to those in phase I or II, with peaks in July. It is apparent that the dam plays an effective role in the sediment interception. From phase I to IV, $\bar{Q}$ varies by a factor of 1.3, while $~\bar{S}$ and $\overline{{Q_{s} }}$ differ by a factor of 3.0 and 3.8, respectively.

**Figure 5:**
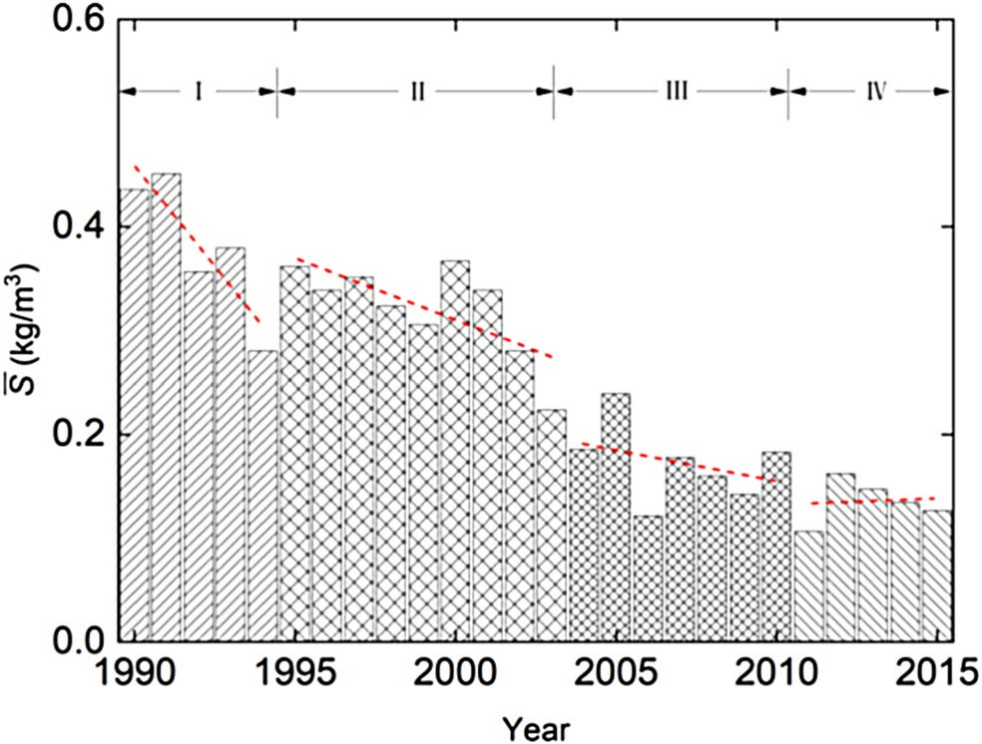
Yearly variations in $\bar{S}$ during the four phases (1990–2015) at Datong station.

Table 3 summarizes the phase-averaged $\bar{Q}$, $\bar{S}$ and $\overline{{T_{s} }}$ values for the four phases. During the phases, $\bar{Q}$ exhibits some fluctuations in magnitude. A study of the river flow discharge records ranging from 1950’s to 1990’s reveals that the differences among the years are natural variations. The reservoir impoundment plays certainly a significant role in flood regulations. Figure 5 shows that the decrease in $\bar{S}$ is, on the whole, sustained over the years. As appears, both $\bar{S}$ and $\overline{{T_{s} }}$ decline dramatically on a yearly or multi-year basis, especially in phases II and III, which is suggestive of the pronounced impacts of the dam on suspended load interception. The role of the flow discharge variations seems to be secondary.

**Table 3:** Phase-averaged flow and sediment in each of the four phases (1990–2015) at Datong station.

Phase	$\bar{Q}$ (m^3^/s)	$\bar{S}$ (kg/m^3^)	$\overline{{T_{s} }}$ (10^8^ t)
I	28,657	0.302	2.729
II	30,654	0.249	2.407
III	26,115	0.144	1.186
IV	26,928	0.119	1.011

#### Sediment sorting

The impoundment of the dam traps the coarse material from its upstream and the fine-grained sediment is released from the reservoir to its downstream^19^. The latter is mainly in the form of suspended and wash loads, which do not easily deposit in the river course^29^. The suspended sediment sorting or grain-size distribution is also an indicator of changes before and after the commissioning.

**Figure 6:**
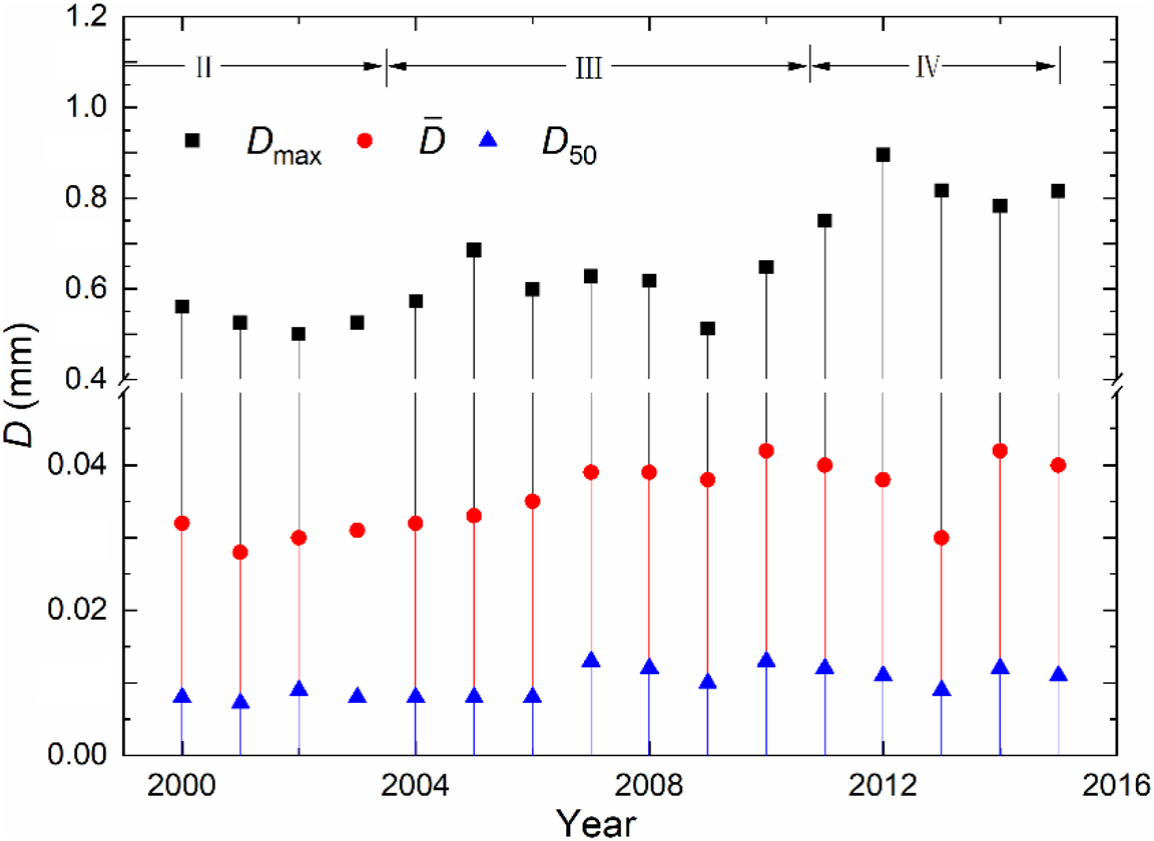
Variations in *D* before and after the start of the impoundment at Datong station (2000–2015).

Figure 6 compares, at Datong station, the changes in particle diameter *D* (mm), expressed in terms of maximum size *D*_max_ (mm), mean size $\bar{D}$ (mm) and medium size *D*_50_ (mm). The diagram is based on the collected monthly data during the 2000‒2015 period, covering phases III and IV and even part of phase II. Before year 2000, the grain-size data is fragmental and therefore excluded. According to the Yangtze sediment bulletin, the sediment sorting analysis changes, as of 2010, from the traditional pipette and sieving method to the laser method. The difference between them is small for the analysis of sand fractions; the latter is a more accurate technique, especially for the sorting of fine-grained sediment^30–32^.

The results show that the variations in neither *D*_50_ nor $\bar{D}$ are significant. However, both sizes exhibit a weakly increasing trend. It is noted that *D*_max_ = ∼ 0.50 mm during 2000–2003 in phase II (before the impoundment); *D*_max_ = ∼ 0.60 mm in phase III; and *D*_max_ = ∼ 0.80 mm in phase IV. The changes, especially in *D*_max_, demonstrates, over the years, an elevated level of particle sizes; the suspended load becomes obviously coarsened, albeit marginally. As aforementioned, after the commissioning, both *S* and *Q*_s_ decline appreciably and become lower than the natural sediment carrying capacity of the flow. As a result, the flow and the riverbed of the sandy material are in competition. The bed scouring leads thus to progressive entrainment of particles into the flow and the sediment sorting changes.

**Figure 7:**
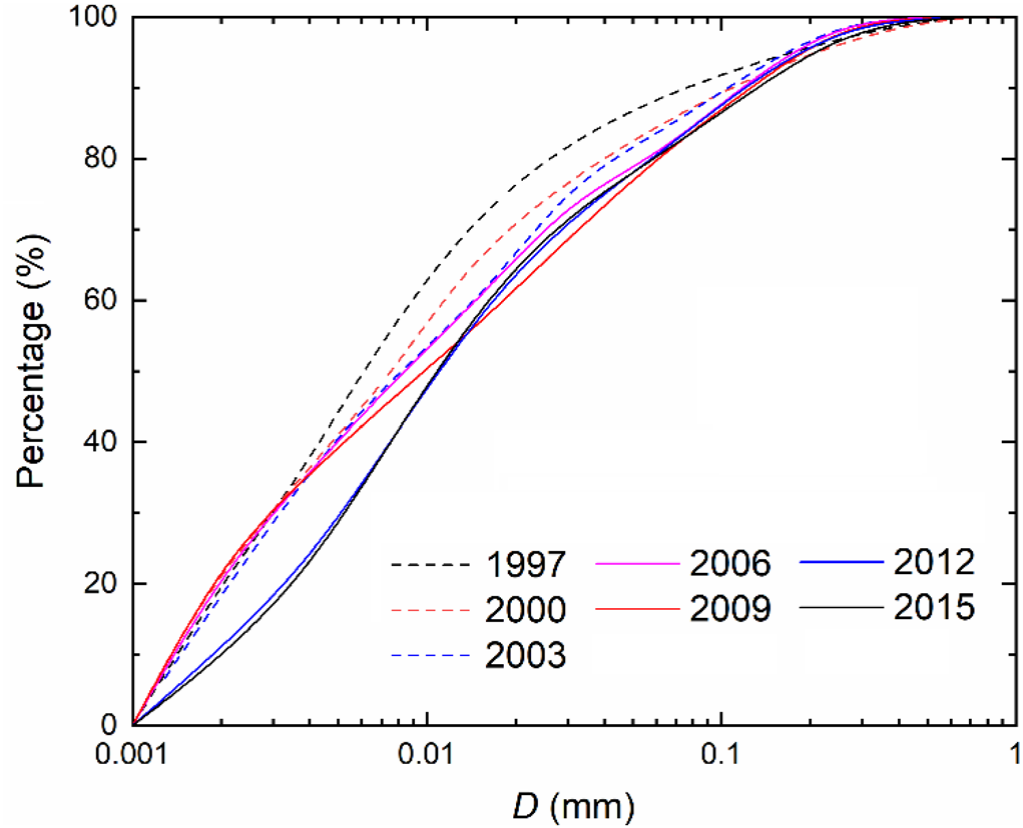
Representative cumulative gradation curves before and after the start of the reservoir impoundment.

To reveal annual variations of particle gradation, Fig. 7 shows the cumulative curves for seven representative years before and after year 2003 that marks the beginning of the impoundment (1997, 2000 and 2003 in phase II, 2006 and 2009 in phase III and 2012 and 2015 in phase IV). *D* is, irrespective of the phases, in a range of 0.001–0.90 mm. In phase II, the *D* ≤ 0.01 mm sediment accounts for ~ 60% of the total fraction, while in phases III and IV it corresponds to 50% and 40%, respectively. The results are in agreement with the data presented in Fig. 6 and imply that the suspended load undergoes a coarsening process, which is indicative of the bed erosion. Yang et al.^7^ reveal that the riverbed sediment coarsens appreciably in the first several 100 km downstream of the TGD.

#### Other aspects of flow and sediment

The dam affects the river flow in three ways, i.e. base water storage, seasonal regulation and evaporation. The dam operation modulates the seasonal flow variations by damping the flood in wet seasons and compensating the discharge in dry seasons. Seasonal flow regulations have only an effect on short-term flow discharges rather than on the annual runoff^8,10,11^. The evaporation is also an issue of concern for water balance downstream, especially on a long-term basis. With the progressive impoundment in phase III, the storage volume and the water-surface area increase. When the reservoir reaches its normal stage, + 175.00 m (phase IV), the active storage capacity is 39.3 × 10^9^ m^3^ and the corresponding surface area is 1.084 × 10^9^ m^2^ (Table 1). The reservoir evaporation amounts to an annual average of 0.3 × 10^9^ m^3^, 0.76% of the active storage volume. Over the first decade of impoundment (2003–2012), the combination of the impoundment and evaporation gives rise to a reduction in the downstream flow by ~ 42.0 × 10^9^ m^3^^10^. Measured at Datong station, the annual decline in the inflow to the study side is ~ 3.82% of the annual runoff ($\overline{{T_{w} }}$) during phase IV, which is compared to ~ 3.40% during phase I.

Owing to the development of several dams and extensive soil conservation measures in the upper river basin, the annual sediment flux into the TGD reservoir has declined in a noticeable manner. The dam also plays a significant role in reduction of sediment in its downstream reaches^7,23,33^. In phases III and IV, the dam traps, on an average, 182 Mt sediment annually, accounting for 80% of the total budget into the reservoir. Estimated at Datong station, this leads to a decline in sediment flux by 65% or 113 Mt per year. Notwithstanding this, some differences exist between wet and dry years. In a wet year as 2010, the figure is ~ 95%, while in dry years as 2006 and 2011, the decline in sediment flux is only 30%^10,20^. All the changes have consequently a bearing on the sediment transport and morphology of the reach concerned.

### Morphological changes after commissioning

Along with the impoundment, the variations in the flow and sediment also lead to morphological changes along the reach. With measured bathymetries in October 1998, October 2010 and September 2015 as a base, the morpho-dynamic responses are compared and the changes are revealed before and after the commissioning. The 1998 bathymetry is 5 years before the impoundment, the 2010 one is at the end of the progressive impoundment, and the 2015 one is after 5 years of normal operation. Sedimentation patterns are now evaluated in terms of both cross-sectional and longitudinal profiles.

#### Cross-channel changes

**Figure 8:**
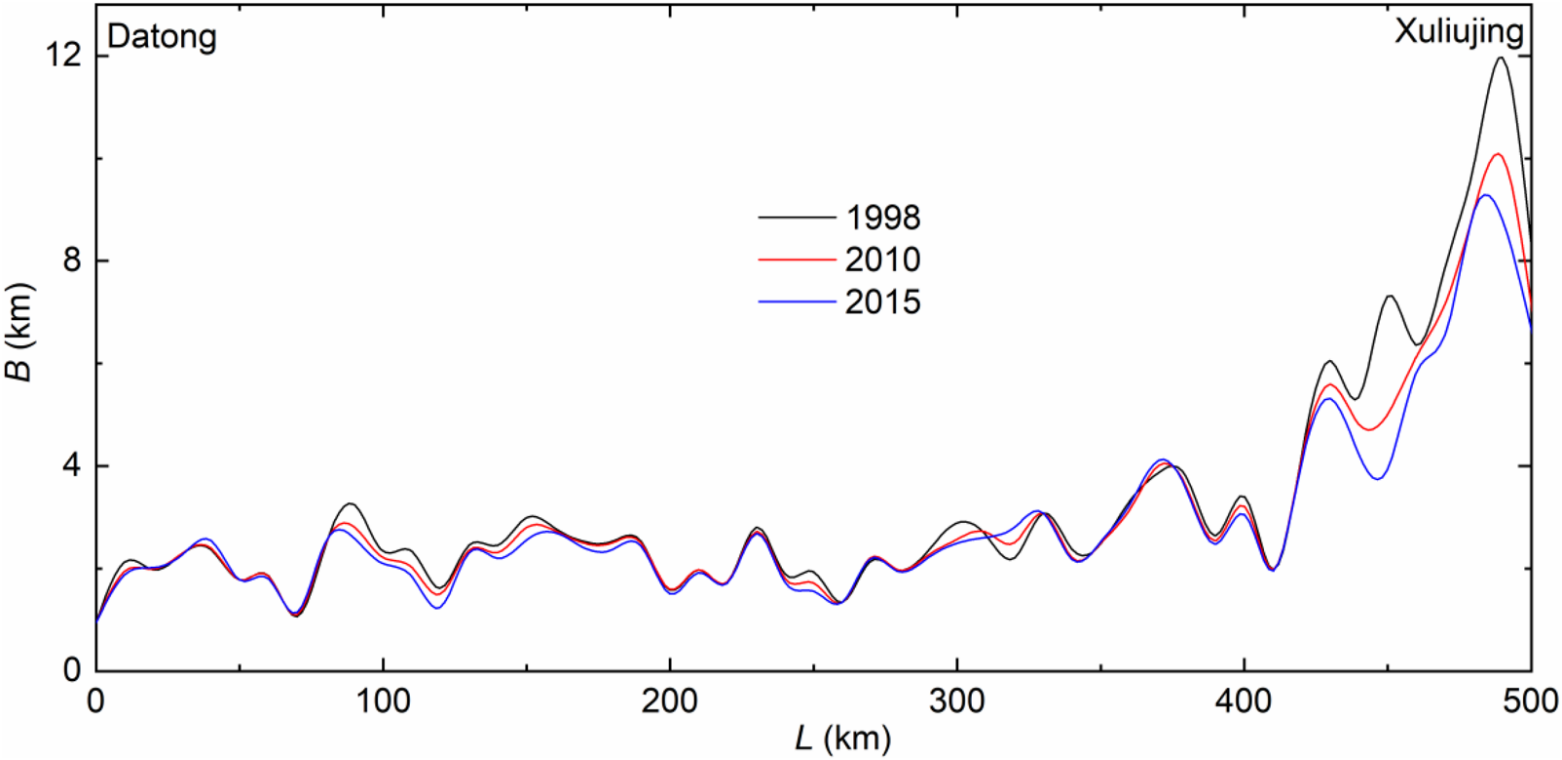
Cross-channel changes along the reach based on the surveys in 1998, 2010 and 2015.

The past decades see cross-channel changes as a result of engineering works (i.e., riparian protection, levees and various bank revetments). Previous studies show that the modifications also give rise to adjustments in the bed morphology^22,34^. Let *B* (m) denote, at a given location, the channel width measured at the mean sea level (0.0 m m.s.l.). The results are presented in Fig. 8.

The field data show that the 500 km river reach narrows, on an average, by 0.375 km during the period. The shrinkage corresponds to 9.5% from 1998 to 2015, which is, in general, attributable to such factors as channel bar growth, reclamation of shoreline, and construction of dikes and revetments^23^. Except for in some sections, the adjustments in width are minor along the upper 420 km. The engineering measures limit the width development in many places. Local widening is mainly due to natural bank erosion processes. Studies made by Xia et al.^22^ and Zhang et al.^24^ demonstrate that the river course upstream of Datong also exhibits insignificant development in width. The major change in *B* occurs in the final 80 km section close to the river mouth, along which the average width changes from 7.462 km in 1998, to 6.544 km in 2010 and to 6.035 km in 2015. This means that the river width is reduced by 1.427 km from 1998 to 2015. Even along this section, the changes are not uniform; some locations are eroded more than others. As compared to the remaining 420 km, the more noticeable reduction is mainly due to enhanced down-cutting, which also augments the flow velocity and reduces flow passage area. That narrowed channels lead to erosional deepening is also observed in many alluvial rivers throughout the world^35,36^.

#### Longitudinal bed changes

Based on the measured bathymetries, the changes in river bed elevation along the reach are assessed. The channel or flow passage volume is a proxy for the changes. To illustrate this, the reach is divided into three segments, delimitated by CS1 and CS2 (Fig. 2). The former is at Wuhu city, 120 km downstream of Datong; the latter is at Zhenjiang city, 180 km upstream of Xuliujing. From 1998 to 2015, the channel volume of each segment is tabulated for comparison (Table 4), corresponding to the space below the mean sea level and above the river bed. With the elapse of time, a larger channel volume of the resulting bathymetry denotes erosional down-cutting, while a smaller one connotes sediment deposition. The volume changes also exhibit an erosion–deposition–erosion pattern. Notice that erosion also takes place upstream of Datong station^22,37^.

**Table 4:** Channel volume changes during 1998–2015.

Segment	Channel volume (10^8^ m^3^)			Difference (c)–(a) (10^8^ m^3^)
	(a) 1998	(b) 2010	(c) 2015	
Upper: Datong–CS1 (120 km)	15.00	16.80	17.31	2.31
Middle: CS1–CS2 (200 km)	34.60	35.15	32.54	− 2.06
Lower: CS2–Xuliujing (180 km)	70.01	70.54	71.26	1.25

**Figure 9:**
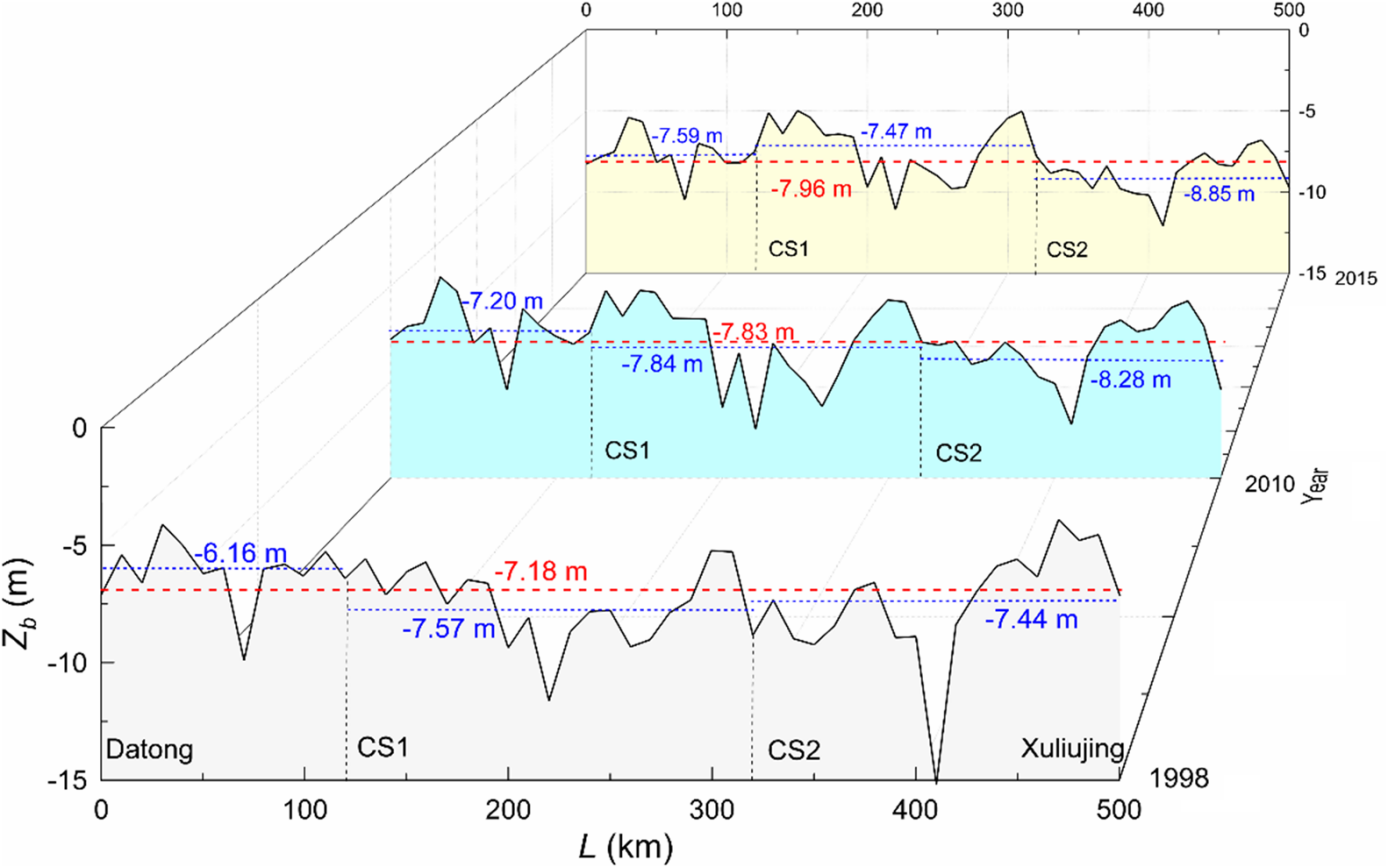
Longitudinal bedform changes of the reach based on the surveys in 1998, 2010 and 2015.

To further quantify the bed changes, longitudinal profiles from the three bathymetries are plotted in Fig. 9, in which *Z*_*b*_ (m m.s.l.) denotes the averaged bed elevation, equal to the ratio of channel volume to *B* × *L*, and *L* (km) is the streamwise distance along the river centerline (*L* = 0 at Datong). Along the 500 km, the reach-averaged *Z*_*b*_ value changes from − 7.18 m in 1998, to − 7.83 m in 2010 and to − 7.96 m in 2015. It shows that the river course undergoes, on the whole, a gradual decline in bed elevation with the elapse of time. However, governed by the river geometry inclusive of bed slope and other factors, deposition also occurs.

For each segment, the *Z*_*b*_ values are also included in Fig. 9. *Z*_*b*_ changes from − 6.16, − 7.20 to − 7.59 m along the upper segment (Datong–CS1), from − 7.57, − 7.84 to − 7.47 m along the middle one (CS1–CS2), and from − 7.44, − 8.28 to − 8.85 m along the lower one (CS2–Xuliujing). Before year 2010, erosional down-cutting occurs obviously along all three segments, which is in agreement with the previous studies by Zheng et al.^23^. Along with the normal operation from 2010, the middle segment shifts, however, from downward scouring to deposition, albeit minor; the erosion continues in the upper and lower segments. The 1998‒2010 period covers phase III and part of phase II. For the middle segment, there are no in-between field data that support if the gradual impoundment slows the erosion and leads to deposition at an earlier time.

For the river course between the TGD and Datong, Yang et al.^14^ reveal that, at a number of locations, there is excessive erosion in response to the declining sediment supply. Similarly, Xia et al.^22^ conclude that the dam operation is the extrinsic cause for the erosion development in the 360-km Jingjiang reach, whose upper end is about 102 km from the dam. The analyses presented here show that erosion extends gradually from Datong to the lower river. The results match the conclusions derived by Zheng et al.^23,25^, in which comparisons are between the bathymetric data from 1998 and 2013. The only drawback of their study is that the interjacent changes during this relatively long period are not unveiled.

In the middle segment, the sediment transported from upstream settles down. Sampled bed materials also show that a layer of 1–3 cm thick mud covers the sandy bed on the top^25^, implying the deposition is likely to be associated with seasonal floods. The tidal effects that are known to be considerable for the Yangtze River vanish further upstream and thus play a minor role in the sediment deposition.

The Xuliujing station is located some 60 km from the river mouth. In the lower segment, the erosion is profoundly affected by the tidal excursions up the river. Attachment of the seaward ebb tides to the river runoff aggravates the erosion, while the suppression of the runoff by the landward flood tides often leads to deposition. The former plays however a dominant role. In this context, a similar phenomenon is also observed in the lower Mississippi River^38^.

In the study area, the topographic changes are affected by the declining sediment budget from upstream. Local human activities are also impact factors^25^. In the recent years, wading projects including bridges and docks have been developed, narrowing the river course and resulting in local erosion. River regulation measures inclusive of construction of embankments limit the width, restrict the river meandering and aggravate the erosion phenomenon. Regular in-channel dredging also contributes to the river bed degradation^39^. Actually, lots of waterway projects (also sand mining and others) are conducted during this period. With the purpose to facilitate the inland navigation in the lower segment, dredging is continually carried out during 2012–2018. Along the central part of the river, the bed elevation is lowered by 2 m; the water depth changes from 10.5 to 12.5 m downstream of Nanjing city. During 2010–2015, the total dredged volume amounts to 4.36 × 10^7^ m^3^^40,41^. In the context, the human activities play a significant role.

## Prediction for near-future changes

With the bathymetrical data from 2010 and 2015, the foregoing analysis reveals, for approximately 5 years’ normal operation (phase IV), the temporal flow and sediment relationship and morphological changes along the reach. However, one question, probably a consequential one in the context, remains unanswered. It is unclear whether or not the river-bed changes have reached a hydro-morphological equilibrium under the impacts of the TGD, i.e. whether the bed morphology will further evolve with time after 2015 and at what pace the change will occur. Practical challenges such as laboratory space, construction costs and flexibility to realize flow conditions limit the possibility to carry out physical hydraulic model tests on such a scale. In view of this, two-dimensional (2D) numerical simulations are performed to help shed light on the near future sediment and morphological changes in the large alluvial river.

### Model setup

The Delft3D program package is a widely used software for solutions of river flow and sedimentation issues^42^. It is adopted here to predict near-future morphological changes. The governing equations are the Navier-Stokes equations (for flow continuity and momentum) and formulas for sediment transport and bed-form deformation. With the sediment trapping in the reservoir, the bed-load flux is insignificantly small and the suspended load accounts for the morphological adjustments of the reach. Its transport is expressed by an advection-diffusion (mass-balance) equation. The bed-form change is determined via the bed stability coefficient and bed resistance. The model is solved with the finite-difference method. For a detailed description of its mathematical formulations (current version 4.04), see the Delft3D website (https://oss.deltares.nl/web/delft3d) and other published results such as Baar et al.^43^ and Rinaldi et al.^44^.

**Figure 10:**
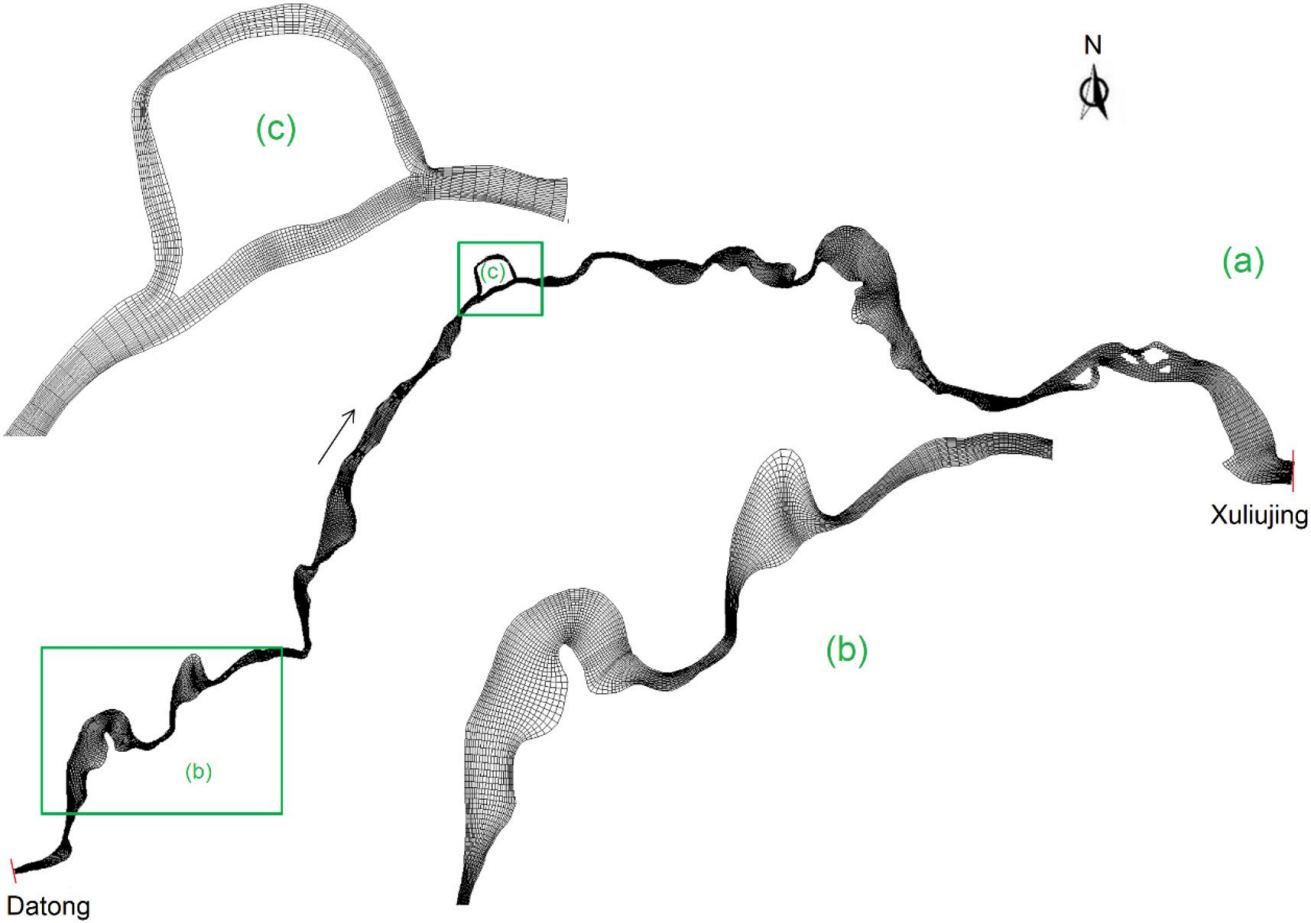
Numerical grid with local enlargements.

The computational domain runs from Datong to Xuliujing, the same as in the field studies (Fig. 1). Due to the appreciable changes in river cross-section including bends, diffluences and confluences a 2D model is set-up. This means that the flow in the vertical direction is treated depth-averaged. Several meshes of varied cell sizes are evaluated so as to ensure grid independent solutions, which are checked through steady-state flow calculations. The along- and cross-channel grid sizes are equal to 30–50 and 40–70 m, respectively (Fig. 10). Small cells are assigned to locations with large flow gradients. The domain is covered by a total of 360,000 cells.

The simulation period stretches over a 15-year period from October 2010 to September 2025. The modeling uses the river bathymetry data from 2010 as initial conditions (Fig. 2). The boundary conditions are defined in terms of both flow and sediment, which are based on the in-situ measurements. The upstream boundary at Datong is specified with the daily-averaged flow discharge and suspended load concentration; the downstream one at Xuliujing is prescribed with the corresponding water-level changes with time. The model runs first to 2015 for model calibration and validation—the numerical results are compared with the recorded water levels at the two stations and the mapped bathymetry. Using the morphological accelerate technique in the software, the 2010–2015 changes are tripled and the simulation extends by 10 years to 2025.

### Sedimentation trends

**Figure 11:**
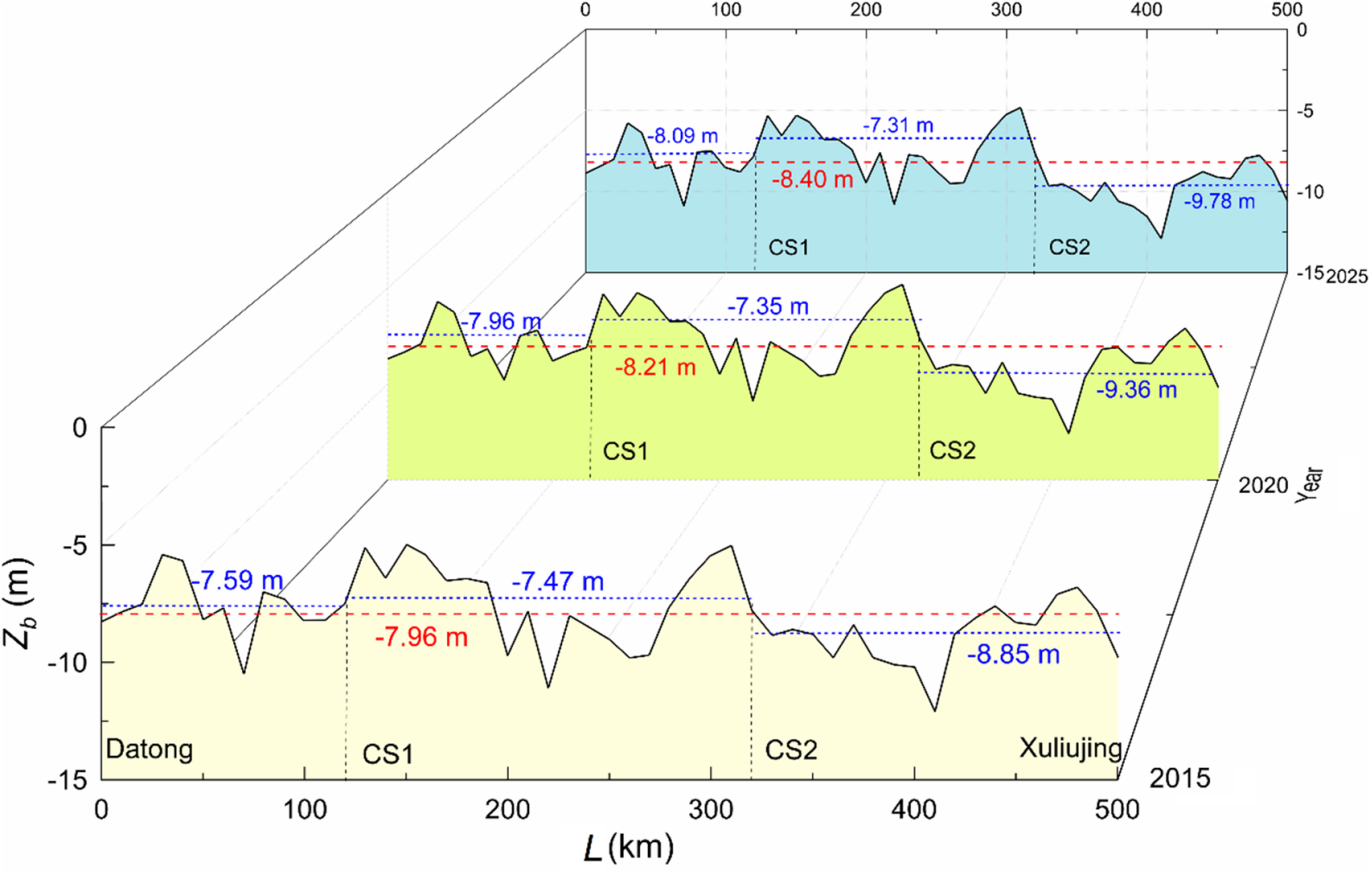
Longitudinal bedform changes of the reach in 2015, 2020 and 2025.

In the light of the 2010 and 2015 bathymetries and the 5-year daily flow and sediment data during October 2010–September 2015, predictions are made to look into the potential morphological changes in the near future. The riverbanks confined by levees and revetments are kept at the same width in the modelling; only downcutting is allowed. Figure 11 plots and compares the longitudinal bed profiles on three occasions in 2015, 2020 and 2025, the latter two of which are the predicted profiles. Along the 500-km reach, the reach-averaged changes are from − 7.96 m in 2015, to − 8.21 m in 2020 and to − 8.40 m in 2025. This means that the river course is expected to deepen further, by 0.44 m during the 10-year period.

**Figure 12:**
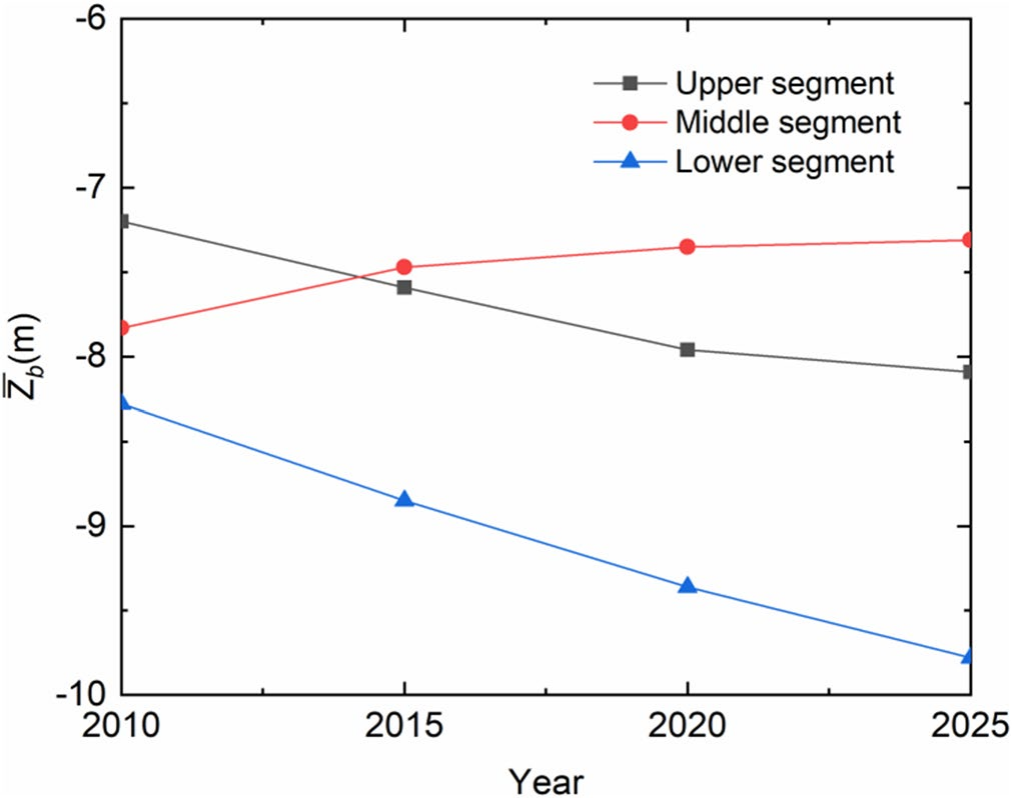
Segment-averaged longitudinal bedform changes during 2010–2025.

The segment-averaged *Z*_*b*_ values are also indicated in Fig. 11. It changes correspondingly from − 7.59, − 7.96 to − 8.09 m in the upper segment, from − 7.47, − 7.35 to − 7.31 m in the middle one, and from − 8.55, − 9.36 to − 9.78 m in the lower one. This implies that, along with the normal reservoir operation, erosion continues in the upper and lower segments; the middle segment incurs gradual deposition. This is suggestive of the fact that the trend that that the reach exhibits during 2010–2015 will remain for at least another 10 years. Figure 12 summarizes the *Z*_*b*_ values of each segment as a function of time. The erosion in the upper segment and the deposition in the middle one do not show any sign of escalation, but slow down instead. The erosion in the lower segment bears however some analogy to the previous years, with an almost linear downward trend.

**Figure 13:**
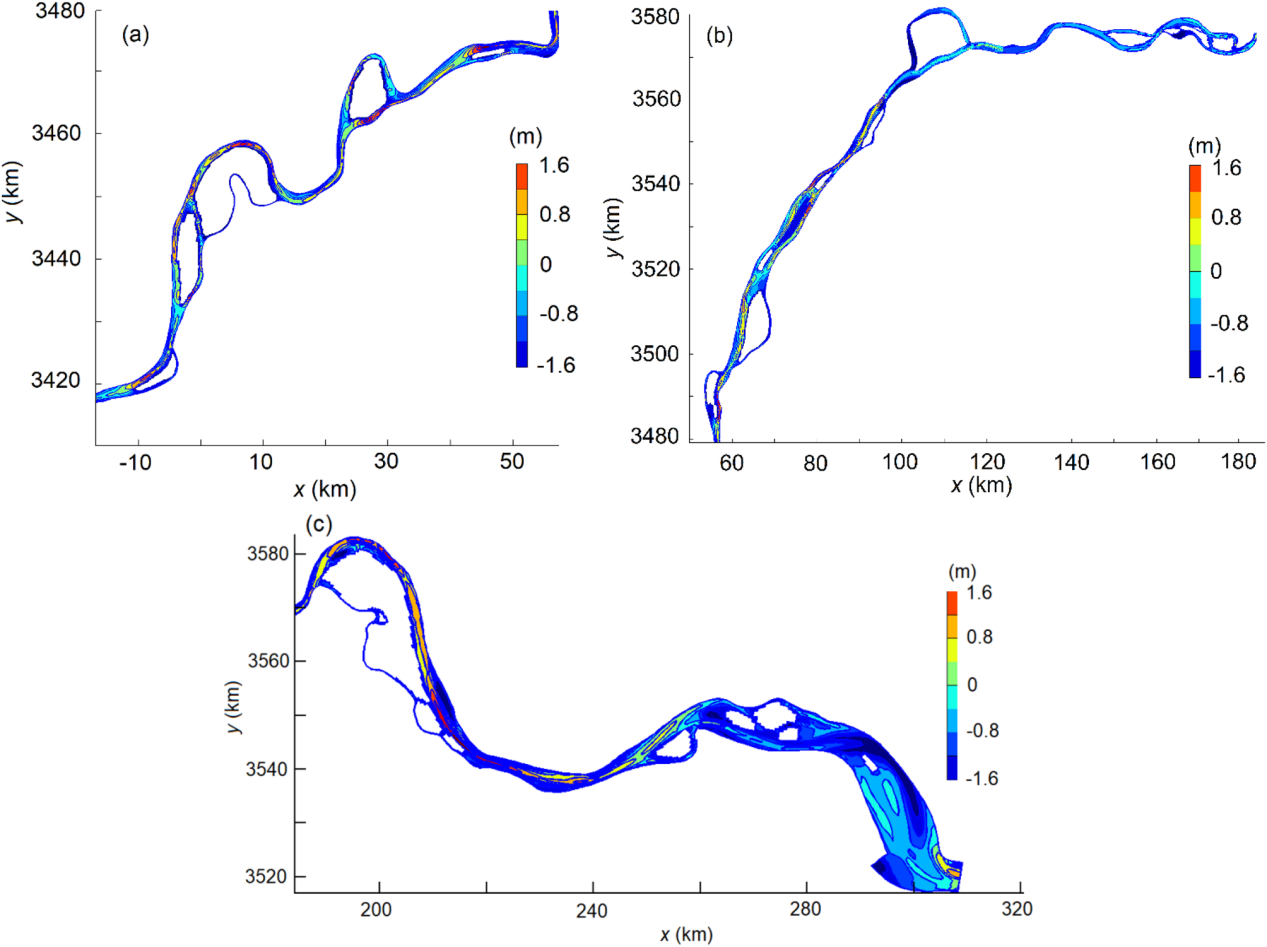
Comparison of bedforms between 2015 and 2025. (**a**) Upper segment (Datong–CS1); (**b**) middle segment (CS1–CS2) and (**c**) lower segment (CS2–Xuliuing).

Being large in width and erodible in an alluvial plain, the river reach is characterized by meanders, braids and an appreciable number of bars and islands. The general pattern of e.g. erosion is also accompanied by local deposition and vice versa. Figure 13 compares, in form of isolines, the differentiated elevations between the 2015 and 2025 bathymetries, calculated by subtracting the latter from the former. A positive number thus denotes erosion and a negative one deposition. (*x*, *y*) refers to a local coordinate system, pointing positively seaward and northward, respectively (Fig. 2). In the upper and lower segments, the average bed elevation falls by 0.50 m and 0.93 m. The scour occurs in the main channel; local erosion depth amounts, at maximum, to 1.60 m at quite a number of locations.

The 200 km middle segment is, on the whole, exposed to gradual deposition. On an average, the bed level uplifts by 0.16 m. In some shore areas, local siltation can be up to 1.25 m thick. The simulations show that the sediment deposition continues to mid-September 2018 and then switches to slight erosion, which takes place first at the upper segment end. In the simulations, the choice of Manning’s roughness coefficient has a bearing on the results, for which sensitivity analyses are made. Within its reasonable range, the shift from deposition to erosion could be one month earlier or later. Nevertheless, no field bathymetry data later than 2015 are available to verify this. The reservoir reaches its full pool level first in October 2010. This suggests that the sedimentation pattern here shifts after approximately 8 years’ full impoundment. By 2025, the erosion has extended some 90 km down the segment. Except for some local severe scours, the erosion depth varies between 0.20 and 0.70 m.

The results demonstrate that, along with the full impoundment, the river reach is still in a state of adjustment. This is contradictory to the hypothesis that scouring further downstream initiated by the TGD is prevented and a new hydro-morphological equilibrium is reached^26^. Surian and Rinaldi^35^ state that impoundment of a dam often gives rise to a substantial reduction in sediment budget to its downstream reaches. The river course downstream then yields to a gradual adaptation to the changes, often with consequential bed erosion. It is a slow process and the time scale may be in the order of decades or even longer.

**Figure 14:**
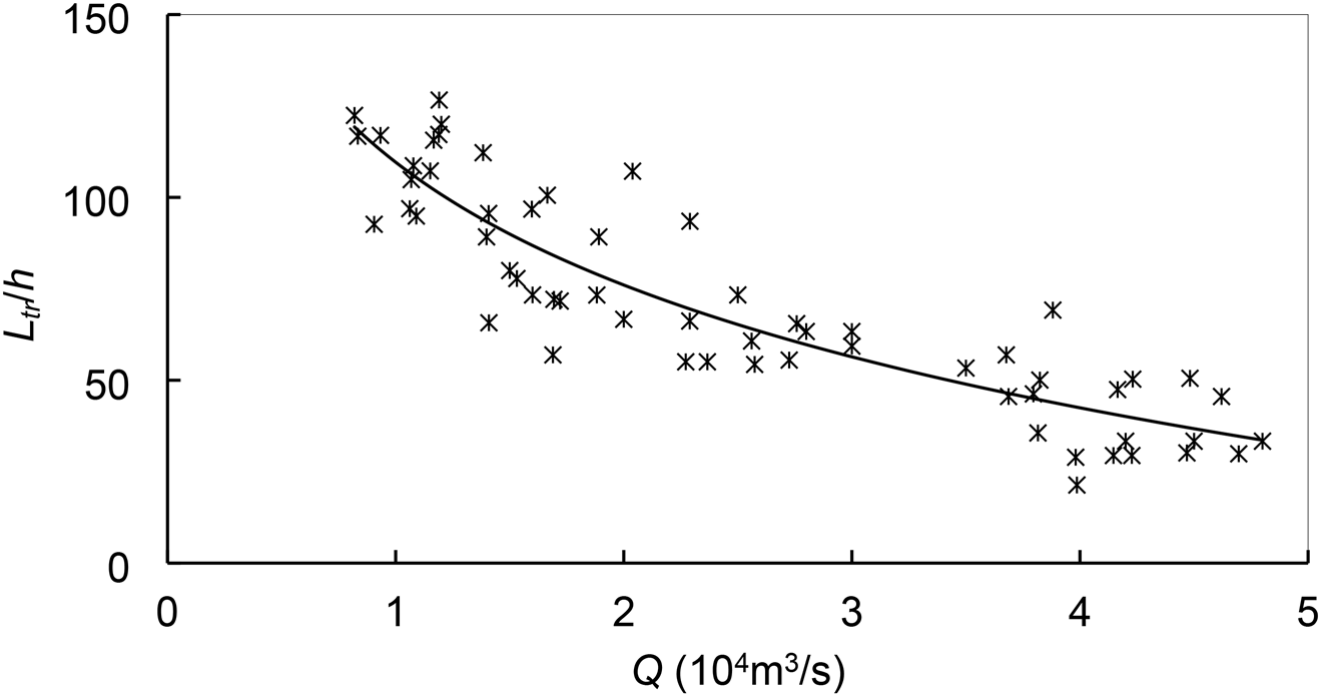
Dependence of *L*_tr_ on *Q* and *h*.

In the lower segment, the trend of continued erosion is integrated with the tidal waves that propagate up and down the river. The segment inclusive of the Xuliujing station is significantly affected^45^. The maximal tidal range *h* (m) amounts to 3.2 m at the end of the examined reach. The interplay with the seaward runoff generates a bi-directional flow. Being a proxy of the tidal effects, let *L*_*tr*_ (m) denote the distance from Xuliujing to tidal reversal (TR) location. Zhang et al.^46^ show that *L*_*tr*_ is mainly governed by *Q* and *h*. Based on the simulations, The *L*_*tr*_/*h* data collapse to a single curve and exhibit a logarithmic change with *Q* (Fig. 14). Depending on *Q* and *h*, *L*_*tr*_ varies between 50 and 350 km. The data are in line with the results from other studies^45,47^. That erosion continues in the lower segment is attributed to the interplay between the tides and the river runoff, with the former being dominant.

The simulated bedforms do not include the impacts of dredging. Along with the rapid social and economic development, the river has become more trafficked than ever. In-channel dredging, which is made at regular intervals to facilitate the navigation, also contributes to the local bed degradation^39^. As shown in Fig. 3, the *T*_s_ and *S* have decreased dramatically in connection with the impoundment. Studies made by Yang et al.^7,14^ reveal that, in the forthcoming decades, an additional decrease in the sediment budget is expected at the dam, which is owing to the dam constructions and effective soil conservation controls in the upper river basin. Over the past decades, large hydropower projects have been developed in the upper reaches of the river^14^. For example, the dam reservoirs at Xiangjiaba and Xiluodu start impounding in October 2012 and May 2013, respectively. They regulate the river flows and intercept the sediment, resulting in abatement in sediment inflow to the Three Gorges reservoir. In view of the declining sediment supply in combination with erodible riverbed, bed downcutting in the 500 km reach is a plausible scenario.

## Conclusions

The Three Gorges represents one of the largest hydropower projects in the world. Exclusive of the preparations, the dam construction begins in 1995 and covers a period of 16 years. It takes about 7 years to attain full impoundment in October 2010. The reservoir operations modulate the sediment transport and have a bearing on the morphology downstream. In the light of field investigations, numerical simulations and a blend of data sources from related publications, the study deals, before and after the commissioning, with the sedimentation changes along the 500 km of the lower river. Based on the 26-year field data, the study assesses the temporal flow and sediment relationship during 1990–2015, and elucidates the changes, both cross-sectional and longitudinal, in morphology. The numerical simulations aim to predict the channel evolution in the near future.

The dam impoundment regulates the seasonal flow discharges and traps the sediment inclusive of bed load. As a result, both the sediment concentration and flux decrease pronouncedly downstream. From the pre-construction to the full impoundment phase, the multi-year averaged maximum flow discharge varies by a factor of 1.3, while the corresponding suspended load concentration and transport rate differ by a factor of 3.0 and 3.8, respectively. The decline in the concentration augments the sediment carrying capacity of the flow and heightens the scouring potential in the riverbed. The bed sediment becomes thus coarser as time elapses, indicative of sediment particle re-sorting.

Irrespective of the project phases, the 120 km upper and 180 km lower segments of the 500 km of the river always suffer from a scouring process. In the former, along with the full-pool impoundment from 2010, the bed level downcutting slows gradually down towards 2025. In the latter, the erosion continues at a constant pace, which is integrated with the tidal actions up the estuary. In the 200 km middle segment, the general erosion switches to slight deposition sometimes during the progressive impoundment and lasts to September 2018. Then erosion begins at its upper end and extends some 90 km down the segment by 2025. The noticeable sediment reduction from upstream is the extrinsic cause for the river-bed erosion.

As of June 2003, the impoundment has resulted in a gradual reduction in sediment supply. The river course downstream sees a process of progressive adjustment to the changes. It seems that the 500 km reach has not yet achieved a hydro-morphological equilibrium and the riverbed down-cutting is going to continue for a period.

The study predicts the general trend of near-future sedimentation and morphological changes. With the 2010–2015 field data as basis, the prediction covers 10 years from 2015 to 2025. As the waterway is huge and complex, featuring appreciable variability in both flow and sediment, more recent and updated field data of bathymetry, flow and sediment are deemed necessary to extend the modeling and make reliable prediction in a longer perspective.

The construction of dams leads to hydraulic fragmentation of a river catchment. A reservoir modulates the river flows and interrupts the sediment transport to its downstream reaches. Soil conservation measures upstream also cut down the sediment flux into the reservoir. As a result, the reduction in sediment budget and the significant morphological adjustments downstream become a fact, which has implications for the society and the economy. It is the intention of this research to provide reference for the study of similar sedimentation issues in alluvial rivers.

## Data Availability

The data that support the findings of the study are available from Yangtze Water Resources Commission, Hydrology and Water Resources Survey Bureau of the Yangtze River Estuary and Yangtze Waterway Bureau. Source data are not provided with this paper.

## Code availability

The software used for the simulations in this study is available at https://oss.deltares.nl/web/delft3d/home.
